# DNA methylation profiles in the blood of newborn term infants born to mothers with obesity

**DOI:** 10.1371/journal.pone.0267946

**Published:** 2022-05-02

**Authors:** Aya Sasaki, Kellie E. Murphy, Laurent Briollais, Patrick O. McGowan, Stephen G. Matthews

**Affiliations:** 1 Department of Physiology, University of Toronto, Toronto, Ontario, Canada; 2 Lunenfeld-Tanenbaum Research Institute, Sinai Health System, Toronto, Ontario, Canada; 3 Department of Obstetrics & Gynecology, Mount Sinai Hospital, University of Toronto, Toronto, Ontario, Canada; 4 Dalla Lana School of Public Health, University of Toronto, Toronto, Ontario, Canada; 5 Department of Psychology, University of Toronto, Toronto, Ontario, Canada; 6 Departments of Biological Sciences and Cell and Systems Biology, University of Toronto, Toronto, Ontario, Canada; McGill University, CANADA

## Abstract

Maternal obesity is an important risk factor for childhood obesity and influences the prevalence of metabolic diseases in offspring. As childhood obesity is influenced by postnatal factors, it is critical to determine whether children born to women with obesity during pregnancy show alterations that are detectable at birth. Epigenetic mechanisms such as DNA methylation modifications have been proposed to mediate prenatal programming. We investigated DNA methylation signatures in male and female infants from mothers with a normal Body Mass Index (BMI 18.5–24.9 kg/m^2^) compared to mothers with obesity (BMI≥30 kg/m^2^). BMI was measured during the first prenatal visit from women recruited into the Ontario Birth Study (OBS) at Mount Sinai Hospital in Toronto, ON, Canada. DNA was extracted from neonatal dried blood spots collected from heel pricks obtained 24 hours after birth at term (total n = 40) from women with a normal BMI and women with obesity matched for parity, age, and neonatal sex. Reduced representation bisulfite sequencing was used to identify genomic loci associated with differentially methylated regions (DMRs) in CpG-dense regions most likely to influence gene regulation. DMRs were predominantly localized to intergenic regions and gene bodies, with only 9% of DMRs localized to promoter regions. Genes associated with DMRs were compared to those from a large publicly available cohort study, the Avon Longitudinal Study of Parents and Children (ALSPAC; total n = 859). Hypergeometric tests revealed a significant overlap in genes associated with DMRs in the OBS and ALSPAC cohorts. *PTPRN2*, a gene involved in insulin secretion, and *MAD1L1*, which plays a role in the cell cycle and tumor suppression, contained DMRs in males and females in both cohorts. In males, KEGG pathway analysis revealed significant overrepresentation of genes involved in endocytosis and pathways in cancer, including *IGF1R*, which was previously shown to respond to diet-induced metabolic stress in animal models and in lymphocytes in the context of childhood obesity. These preliminary findings are consistent with Developmental Origins of Health and Disease paradigm, which posits that adverse prenatal exposures set developmental health trajectories.

## Introduction

Obesity is a risk factor for many chronic diseases. Rates of obesity are increasing in adults and children; over 600 million adults and 100 million children worldwide had obesity in 2015 [1]. Rates of obesity correlate among family members, and mothers with obesity tend to have children with obesity [2, 3]. The children of mothers with obesity early in pregnancy have more than twice the risk of obesity between ages 2 and 4 [4], and adiposity at birth predicts adiposity at age 8 [5]. In animal studies, maternal obesity has been shown to result in a number of poor health indicators in offspring, including increased adiposity, insulin resistance, and hyperphasia [6] with sex differences in adult offspring that are independent of high caloric diet consumption in postnatal life [6, 7]. Similar to studies in animal models, findings in humans indicate that maternal obesity influences the prevalence of cardiovascular and metabolic diseases in offspring in a sex-specific manner [8]. In this context, women appear at greater risk of metabolic disease than men, due to in part to the influence of sex-specific genetic and steroid hormone regulatory mechanisms in development [9, 10]. Adverse prenatal exposures are thus recognized as important components of the Developmental Origins of Health and Disease (DOHaD) paradigm, which proposes that exposures in early life set developmental health trajectories [11]. As the prevalence of childhood obesity is also influenced by postnatal factors, including diet and physical activity, it is critical to determine whether the children born to women with obesity during pregnancy show alterations that are detectable at birth.

Epigenetic modifications are proposed mechanisms of prenatal programming. DNA methylation modifications at CpG dinucleotides have been most extensively studied in this context. Specific DNA methylation modifications are required for normal development. They are associated with several key processes, including genomic imprinting and the risk of non-communicable diseases [12]. To our knowledge, only four studies have examined the association between maternal obesity and DNA methylation modifications in whole cord blood [13–16], though modifications associated with offspring sex were not reported, despite known sex differences in prenatal effects on growth and adiposity [8]. For example, male sex hormone testosterone increases in embryonic life [17], with a testosterone surge in the second trimester [18]. The number of X chromosomes alone affects adiposity, where dosage of the X chromosome leads to higher adiposity postnatally in mice [19]. Also, the timing of dynamic DNA modifications such as demethylation is earlier in the paternal than in the maternal genome [20]. We hypothesized that children born to women with obesity during pregnancy show alterations in DNA methylation in whole blood that are detectable at birth and are sex-specific.

In the present study, samples were obtained from an ongoing cohort study, the Ontario Birth Study (OBS), based on the availability of non-self reported maternal BMI measured in the first visit of pregnancy care (~12 weeks of pregnancy). Mothers with obesity and normal weight mothers were matched based on maternal age, parity, and infant sex. Strict exclusion criteria included pregnancy complications previously shown to influence DNA methylation signatures, including diabetes [21, 22] and preterm birth [23], chronic use of glucocorticoids (as in asthma or collagen vascular disease) and treatment with glucocorticoids during pregnancy [24] to minimize the influence of these disease states and associated pharmacological treatments. In the resulting study, we examined genomic loci associated with differential methylation as a function of maternal obesity status in dried blood spots collected at term from neonates of both sexes. We used reduced representation bisulfite sequencing (RRBS) to quantitatively profile DNA methylation modifications of CpG dense regions, where DNA methylation is most likely to influence gene regulation, including CpG islands, promoters, gene bodies, and intergenic regions [25]. We also compared gene-specific differential methylation associated with maternal obesity to those in an existing cohort, the Avon Longitudinal Study of Parents and Children (ALSPAC). Data from this cohort were obtained by 450K microarray, which focuses predominantly on CpGs in RefSeq genes (NM and NR; 98.9% of all UCSC RefGenes) [26]. Therefore, the focus of this comparison was on genes and gene pathways associated with differential methylation identified by our primary analysis.

## Materials and methods

### Subjects and blood samples

Subjects included in this study were selected from women recruited into the Ontario Birth Study (OBS) at Mount Sinai Hospital in Toronto, ON, Canada [27]. This study was approved by the Mount Sinai Hospital Research Ethics Board (MSH REB# 17-0090-E) and the University of Toronto Research Ethics Boards. All persons gave their informed consent prior to their inclusion in the study. Informed consent was obtained from the mothers and from the mothers on behalf of the minors included in this study. Blood samples were collected on Guthrie cards (Whatman 903) by heel prick 24 hours after birth as a part of routine neonatal screening, with an additional card taken for research purposes. The samples were air-dried for at least 4 hours at room temperature and stored at -80°C. At the time of subject selection, women with obesity were defined as BMI ≥ 30 kg/m^2^ based on their medical records taken during the first visit of pregnancy care (i.e., ~12 weeks of pregnancy) compared to control women with BMI 18.5–24.9 kg/m^2^ who were matched by maternal age (within 5 years), parity (multiparous or nulliparous), and infant sex to control for factors associated with birth weight [28, 29]. All pregnancies were singleton and delivered at term (≥ 37 weeks). Chronic use of glucocorticoids (e.g., asthma or collagen vascular disease), treatment with glucocorticoids during pregnancy, diabetes, gestational diabetes, and hypertension were exclusion criteria. The stringent inclusion criteria led to the selection of 20 neonatal dried blood spots from infants born to mothers with obesity and 20 neonatal dried blood spots from infants born to women with a normal weight with equal numbers of male (n = 10/obese and n = 10/non-obese) and females (n = 10/obese and n = 10 non-obese) in each condition from the OBS cohort (total n = 40).

### Reduced representation bisulfite sequencing (RRBS)

DNA was extracted from the blood spots using methods described previously [30] with some modifications [31]. Briefly, DNA was extracted from small pieces cut from ½ of a blood spot using Proteinase K lysis and following the manufacturer’s protocols (QIAamp DNA Blood Mini Kit (Qiagen: Cat. #51104)). Only samples with DNA Integrity Numbers (DINS) above 7 were included in the study. We generated RRBS libraries using 100ng dsDNA using MspI restriction enzyme digestion and size selection (RRBS Methyl-Seq System 1–16 (Ovation: Part # 0353)). The RRBS libraries were then sequenced in multiplexes of 10 samples, using a NextSeq500 (Illumina) with a 75bp single end read length (Donnelly Sequencing Centre, University of Toronto, Toronto, ON, Canada).

### Differentially methylated regions (DMRs) analysis

Adaptor sequences and low quality reads (q < 30) were trimmed using Trim Galore (https://www.bioinformatics.babraham.ac.uk/projects/trim_galore/) followed by filtering with NuGEN script, as previously described [31]. Alignment and sorting were performed using Bismark [32] and Samtools [33], respectively. DMRs associated with maternal BMI were investigated using the R package methylKit version 1.14.2 [34]. Read counts below 5x and greater than 99.9% of coverage in each sample were discarded to avoid reads showing PCR bias. Default settings in methylKit were used to calculate the bisulfite conversion rate and the reproducibility of the data [34]. MethylKit tiles the genome into non-overlapping Differentially Methylated Regions (DMRs) of 1kb from a given CpG site. We ensured at least 5X reads per CpG site in each sample, as calling significant DMRs has been shown to be sufficient with 5x reads [35, 36]. P-values for each DMR were calculated using the Fisher’s exact test and then adjusted for multiple testing by calculating Benjamini-Hochberg false discovery rate (FDR) corrected q-values using the SLIM method [37]. We considered regions to be DMRs if they were statistically significant at an FDR < 0.05, contained at least 5 CpG sites and had an absolute difference in DNA methylation that was greater than 5%. DNA methylation differences of at least 5% per DMR have been used in several previous studies, including by our group, as a sensitive approach to detect changes more likely to have biological significance [38–42]. Gene annotation was performed using CompEpitool [43] and human genome assembly hg19.

**Fig 1:**
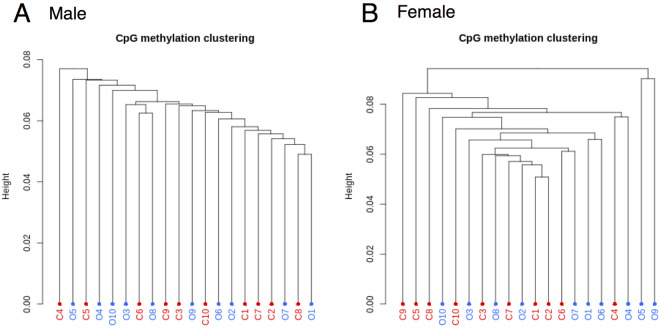
Hierarchical clustering of samples for males (A) and females (B) in the OBS discovery cohort. C: neonates of non-obese control mothers (n = 10/sex), O: neonates of mothers with obesity (n = 10/sex).

For all reduced representation bisulfite sequencing (RRBS) samples, the bisulfite conversion rate was > 99%. S1 Fig shows a histogram of percent methylation for each sample showing a typical percent methylation histogram with peaks on both ends representing the expected distribution of unmethylated and methylated cytosines using methylKit [34]. As shown in S1 Table, the samples showed high correlations for all pair-wise comparisons (>0.8), demonstrating a high degree of reproducibility in the RRBS dataset [34, 44]. In addition, to enable analyses that were representative of group differences rather than skewing by some samples, we filtered for CpG sites that contained at least 5X reads for each subject [45]. This led to over 1.9 million and 2 million CpG sites for analyses in samples from males and females, respectively. We then performed hierarchical clustering of the samples to examine the similarity in methylation profiles overall. Hierarchical clustering of DNA methylation showed that there was neither grouping by condition (obese, non-obese) nor outliers within each sex (Fig 1).

### ALSPAC comparison cohort

Due to the nature of our discovery cohort, which is part of a pilot study, we also examined epigenetic data from the Avon Longitudinal Study of Parents and Children (ALSPAC) cohort study to compare differentially methylated genes between cohorts [46, 47]. Women in Avon, UK, during the period of 1^st^ April 1991 and 31^st^ December 1992 were enrolled in the study in early pregnancy, and samples from 14,610 live births were obtained, in accordance with ethical approval from the ALSPAC Ethics and Law Committee and the local research ethics committees. All participants provided informed consent, following ALSPAC Ethics and Law Committee recommendations (see [48] for full data details). There was no involvement of patients or the public in the decision to perform or report this research. In addition, 1018 ALSPAC mother-offspring pairs participated in the Accessible Resource for Integrated Epigenomics Studies (ARIES), which used Illumina Infinium 450k methylation arrays [49].

The same inclusion and exclusion criteria used in the OBS cohort above were applied. There was no difference between obese and non-obese groups for the variables listed in Table 1 for the OBS, except their BMI was taken from pre-pregnancy measures. Samples from the ARIES cohort were derived from cord blood obtained at delivery (n = 1,127 newborn babies). A total of 444 male neonates (n = 16/obese and n = 428/non-obese) and 415 female neonates (n = 13/obese and n = 402/non-obese) for whom we had information on their mother’s pre-pregnancy BMI, were included in our analyses (total n = 859).

**Table 1:** OBS and ALSPAC subject demographics.

	OBS			ALSPAC		
	Obese	Control	P value	Obese	Control	P value
# of Infants (male/female)	20 (10/10)	20 (10/10)		29 (16/13)	830 (428/402)	
Maternal BMI	36.2 ± 4.8	21.6 ± 1.5	< .01	34.7 ± 4.1	22.2 ± 2.4	< 10^−16^
Maternal age (years)	33.3 ± 3.5	32.8 ± 2.9	n.s.	29.1 ± 4.3	29.7 ± 4.4	n.s.
Gestational age (weeks)	39.8 ± 1.0	39.5 ± 1.3	n.s.	39.6 ± 1.0	39.6 ± 1.5	n.s.
Parity (0/1)	14/6	14/6	n.s.	N/A	N/A	
Birthweight	3571.9 ± 410.6	3537.7 ± 466.2	n.s.	3683.0 ± 541.1	3485.0 ± 457.1	n.s.
Vaginal/C-Section	10/10	15/5	n.s.	<5/>24	45/583	n.s.
(male, female)	(4/6, 6/4)	(8/2, 7/3)	n.s.	N/A	N/A	
Medical History (male/female)						
Depression*	4 (2/2)	1 (1/0)		N/A	N/A	
Anxiety*	2 (2/0)	2 (1/1)		N/A	N/A	
Asthma*	0 (0/0)	3 (2/1)		N/A	N/A	
Data are presented as mean ± s.d. *not medicated Medical histories are not mutually exclusive Mode of delivery is indicated, where data are available P values were calculated by Chi square test for parity and mode of delivery and t-test for other variables n.s.: not significant N/A: not available						

RRBS involves restriction enzyme digestion to enrich for areas of the genome with a high CpG content, whereas in 450K arrays, the coverage per gene has a wide range from one to over 1000 CpG sites [50]. As a result, different R packages optimized for identifying DMRs were used following RRBS and array analysis. Preprocessing, filtering of low quality probes, and normalization of 450K Illumina microarray data were performed using the minfi package, version 1.40.0 [51]. DMRs associated with maternal BMI were investigated using the R package DMRcate 2.0.7 [52]. DMRcate fits a limma linear model, adjusted using an empirical Bayes procedure, for each individual CpG site within loci of 1000bp. Default smoothing parameters across individual CpGs within these loci were used, specifically a Gaussian kernel smoother with a bandwidth *λ =* 1000bp and scaling factor C = 2, with each DMR containing at least 2 CpGs, to account for relative sparsity of coverage by the 450K array in some genomic regions (i.e., intergenic regions) [52]. The resulting kernel-weighted model was compared to the null model using the Satterthwaite method [53] to enable adjustment for multiple testing by FDR. Maximum beta fold-change values per DMR were converted to percentage to summarize effect sizes. As with the sequencing analysis above, DMRs were considered significant if the FDR p < 0.05 with a methylation difference greater than 5% based on the maximum differential methylation value within the DMR. Gene annotation was performed using CompEpitool [43] and the human genome assembly hg19. Hypergeometric tests in R were used to examine the overlap in the number of genes associated with DMRs between the OBS and ALSPAC cohorts.

### Gene pathway enrichment analysis

The lists of differentially methylated genes identified by the DMR analyses were explored using MsigDB, a widely used comprehensive database for gene set enrichment analysis [54]. The enrichment analysis was performed using gene sets derived from both the Kyoto Encyclopedia of Genes and Genomes (KEGG) and the biological Gene Ontology (GO) databases, with significant enrichment defined by FDR p < 0.05 using the default background gene set. These tools were used to aid in the interpretation of the biological meaning behind the list of genes associated with DMRs.

## Results

Subject demographics are listed in Table 1. There were no differences in incidence of (non-matched) pregnancy complications or other aspects of health-related medical history between the two groups except for maternal BMI which, as expected, was significantly higher in the obese group for both the OBS and ALSPAC cohorts. As there were no differences in incidence of (non-matched) pregnancy complications or other aspects of health-related medical history between the two groups, these factors were not included as covariates.

**Fig 2:**
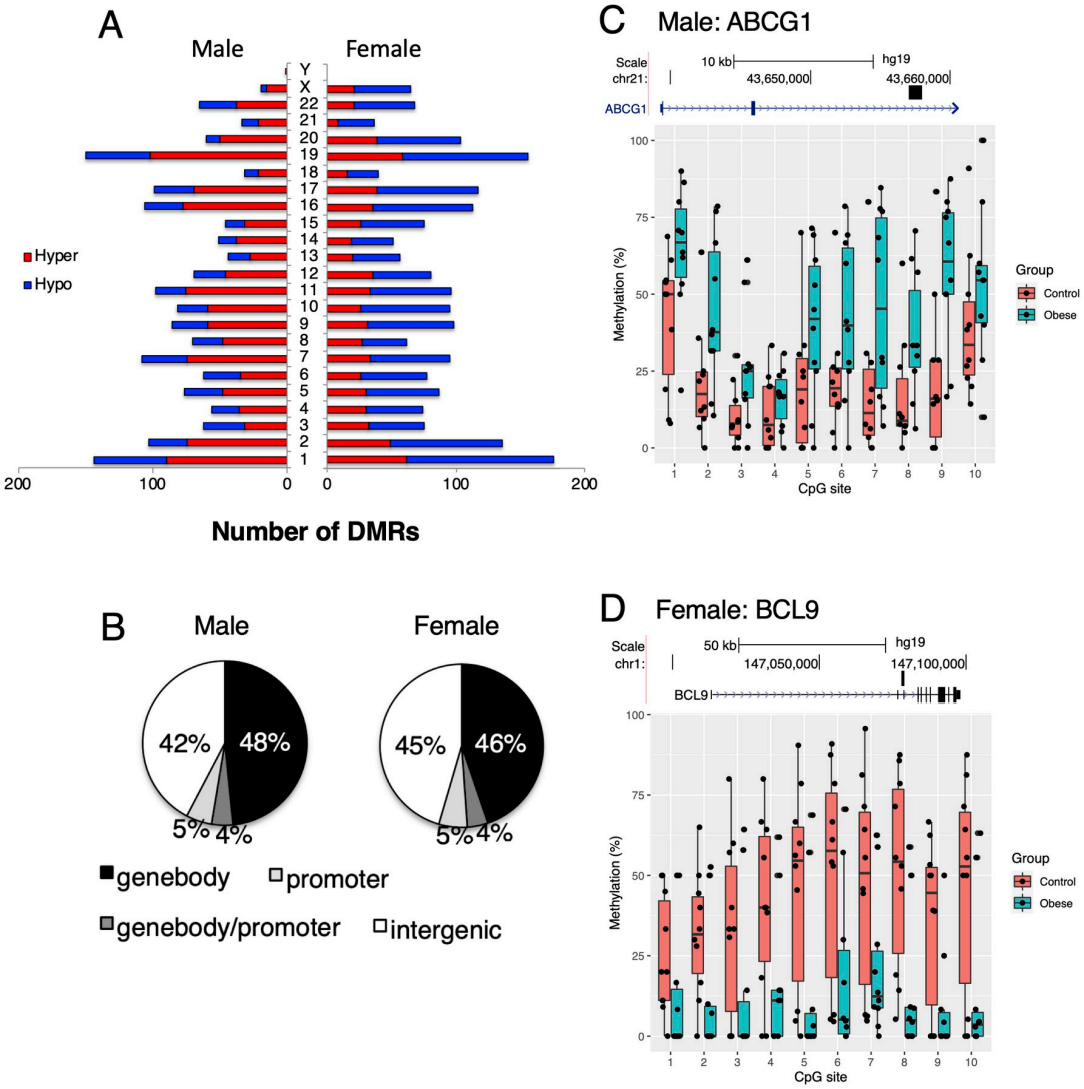
Overview of differentially methylated regions in the OBS discovery cohort. (A) Number of DMRs in relation to chromosomal locations that are either hypermethylated (red) or hypomethylated (blue) in male and female neonates born to mothers with obesity compared to controls. (B) Proportion of DMRs in relation to genetic regions. (C) Representative top DMR in male neonates on the *ABCG1* gene showing methylation percentage in individual CpG sites within the DMR. (D) Representative top DMR in female neonates on the *BCL9* gene showing methylation percentage in individual CpG sites within the DMR. The genomic track shows the gene location starting from the first exon and the DMR location. Hypermethylation refers to greater DNA methylation among neonates from mothers with obesity compared to neonates from mothers with a normal BMI. Hypomethylation refers to lesser DNA methylation among neonates from mothers with obesity compared to neonates from mothers with a normal BMI.

DMRs associated with maternal obesity were found localized to all autosomes as well as in allosomes corresponding to the sex of the offspring (Fig 2A). In male neonates, we identified 1725 differentially methylated regions (DMRs; FDR < 0.05) 1173 regions or 68% were hypermethylated (i.e. greater DNA methylation among neonates from mothers with obesity compared to neonates from mothers with a normal BMI). Five hundred and fifty-two (552) regions or 32% were hypomethylated in male neonates of the mothers with obesity compared to male neonates from mothers with a normal BMI. In female neonates, we identified 2028 DMRs; 710 regions or 35% were hypermethylated and 1318 regions or 65% were hypomethylated compared to females from mothers with a normal BMI. The DMRs identified in male and female neonates were located in genic as well as intergenic loci. Similar numbers of DMRs were localized to intergenic regions in males and females (42% and 45% of all DMRs, respectively). In both sexes, the majority of genic DMRs were found in gene bodies (48% in males and 46% in females). Approximately 9% localized to promoter regions in both sexes (Fig 2B). Examples showing the top differentially methylated DMRs associated with genes in males and females are provided in Fig 2C and 2D. The DMR showing the greatest DNA methylation difference in male neonates was *ATP Binding Cassette Subfamily G Member 1* (*ABCG1*). In female neonates, the top DMRs by percent methylation differences was *B-cell CLL/lymphoma 9 protein* (*BCL9*). The full list of gene-annotated DMRs, sorted by absolute methylation difference in percentage, is shown in S2 Table.

**Fig 3:**
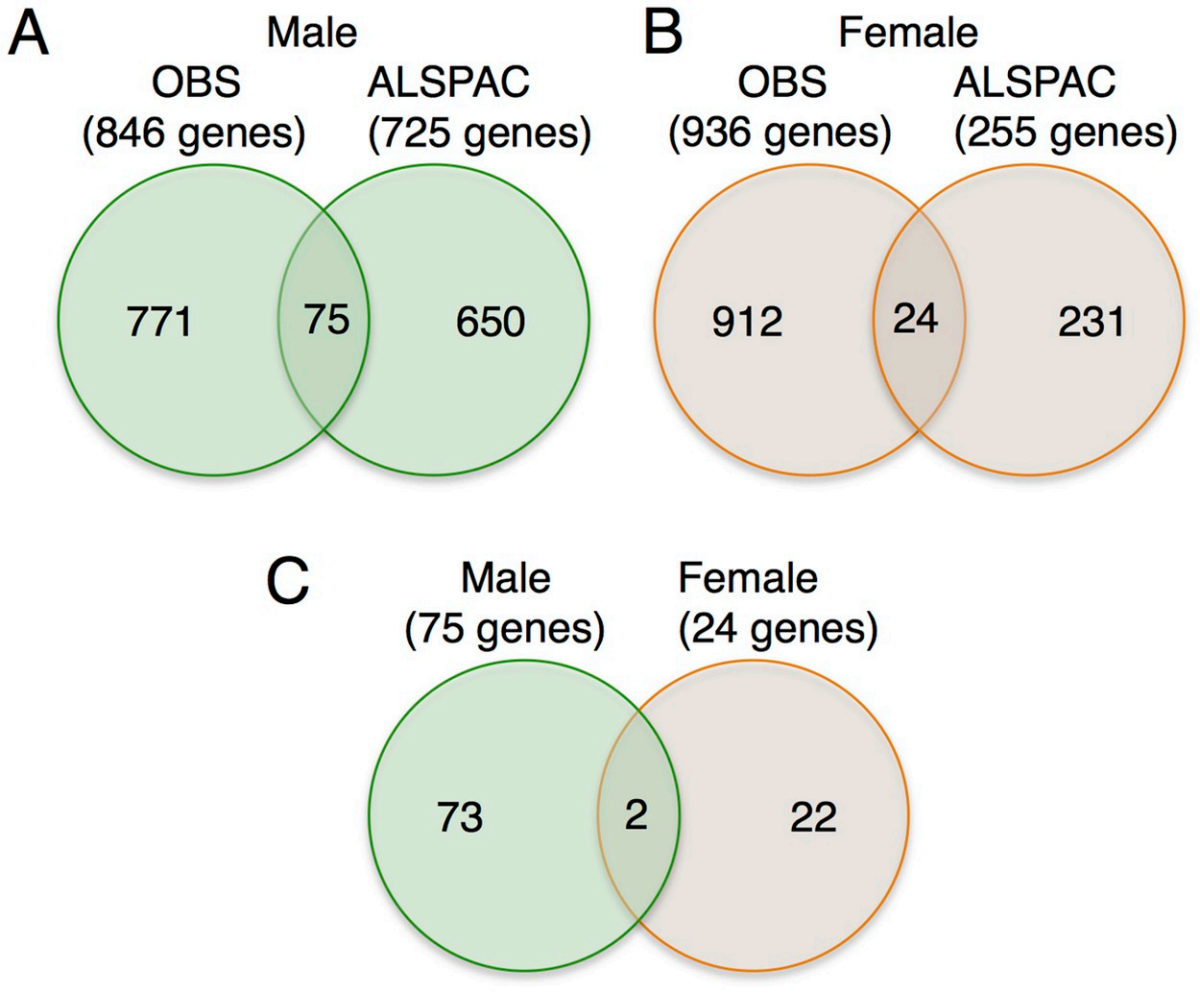
Annotation of differentially methylated regions for the OBS and ALSPAC cohorts. The numbers of genes associated with DMRs for (A) male and (B) female neonates. (C) The number of genes for each sex for genes that replicated across both cohorts. The total number of genes associated with DMRs for each sex are shown in parentheses.

Next, we compared the genes associated with DMRs in the OBS cohort to those of an available population-based cohort, the ALSPAC. The same inclusion and exclusion criteria used in the OBS cohort above were applied. There was no difference between obese and non-obese groups for the variables listed in Table 1 for the OBS, except that BMI was taken from pre-pregnancy measures. In the OBS cohort, male neonates, there were 846 genes associated with DMRs in male neonates and 936 genes associated with DMRs in female neonates (FDRs < 0.05: S2 Table). There were 266 genes common between males and females in the OBS cohort (S2 Table). In the ALSPAC cohort, there were 725 genes associated with DMRs in male neonates and in female neonates, there were 255 genes associated with DMRs (FDRs < 0.05; S3 Table). There were 22 genes common between males and females in the ALSPAC cohort (S3 Table). Among the sex-specific genes, there were 75 genes in common in male neonates and 24 genes in common in female neonates across both cohorts, with 2 genes common to both cohorts and sexes (Fig 3C). Hypergeometric tests revealed significant overlaps in genes associated with DMRs across both cohorts for each sex (P < 10^−12^ for males and P < 0.001 for females).

Table 2 shows a list of top genes containing DMRs in both OBS and ALSPAC cohorts, rank-ordered by the total number of DMRs across both cohorts. *PTPRN2* (Protein Tyrosine Phosphatase Receptor Type N2) and *MAD1L1* (Mitotic Arrest Deficient 1 Like 1) contained DMRs associated with maternal obesity in both sexes in both cohorts. There were 4 and 2 DMRs in *PTPRN2* in OBS (methylation difference -12.5 to 6.8%) and ALSPAC (methylation difference 6.0 to 6.3%) male neonates, respectively. In female neonates, *PTPRN2* had 5 and 3 DMRs in OBS (methylation difference -12.8 to 10.0%) and ALSPAC (methylation difference 5.9 to 6.7%) respectively. For *MAD1L1*, there were 3 and 1 DMRs in OBS (methylation difference 5.7 to 6.2%) and ALSPAC (methylation difference 8.4%) male neonates, respectively. In female neonates, *MAD1L1* exhibited 1 and 1 DMRs in OBS (methylation difference -5.3%) and ALSPAC (methylation difference 6.8%), respectively. Sex-specific genes in common across both cohorts included the imprinted genes *GABRB3* (Gamma-aminobutyric acid receptor subunit beta-3) and *PRDM16* (PR/SET Domain 16) in male neonates. S4 Table shows the full list of genes containing DMRs in common between the OBS and ALSPAC cohorts.

**Table 2:** Top 10 genes associated with differential methylation in neonates, common to the OBS and ALSPAC cohorts. In some instances, multiple DMRs were associated with a given gene therefore a range of methylation difference percentage is provided. Genes with at least three DMRs are shown. The genes in bold were common to both males and females.

Male								
		OBS Meth Diff %			ALS Meth Diff %			
Gene	Sum of DMRs (OBS/ALS)	Range min	Range max	[Avg]	Range min	Range max	[Avg]	Function
CBFA2T3	6 (2/4)	-6.2	5.4	5.8	-5.2	7.8	6.4	Immune function
**PTPRN2**	6 (4/2)	-12.5	6.8	7.6	6.0	6.3	6.2	Insulin secretion
TCERG1L	5 (4/1)	5.0	14.5	8.6	8.2	8.2	8.2	Transcription
ABL1	4 (3/1)	5.7	8.9	7.0	6.6	6.6	6.6	Protooncogene
AGAP1	4 (3/1)	-7.0	10.3	8.3	-7.9	-7.9	7.9	GTP binding
**MAD1L1**	4 (3/1)	5.7	6.2	6.0	8.4	8.4	8.4	Cell cycle
PCDHGA1	4 (1/3)	7.4	7.4	7.4	6.1	6.7	6.3	Cell adhesion
PRDM16	4 (1/3)	5.6	5.6	5.6	5.9	7.2	6.6	Immune function
RPTOR	4 (3/1)	5.4	6.7	6.2	6.6	6.6	6.6	Cell growth
TPPP	4 (2/2)	-6.7	7.0	6.8	-7.1	8.9	8.0	Myelination
**Female**								
**PTPRN2**	8 (5/3)	-12.8	10.0	8.7	5.9	6.7	6.3	Insulin secretion
BOLA2	3 (2/1)	7.0	8.2	7.6	-9.3	-9.3	9.3	Iron maturation
GALNT9	3 (2/1)	5.3	7.6	6.4	9.6	9.6	9.6	Oligosaccharide
PAX7	3 (2/1)	-7.1	-5.3	6.2	-5.9	-5.9	5.9	Fetal development
RAP1GAP2	3 (2/1)	-9.1	7.5	8.3	8.4	8.4	8.4	Immune system
SEC14L1	3 (2/1)	8.6	9.4	9.0	9.9	9.9	9.9	Immune system

We used gene set functional analysis to biologically contextualize interactions between genes in common between cohorts significantly associated with maternal obesity for each sex. In males, KEGG analysis showed significant enrichment in endocytosis and pathways in cancer (FDRs<0.05). In addition, 33 biological GO pathways in common in both cohorts were significantly enriched in male neonates, primarily involving processes related to cellular signaling, immune system function and development (FDRs <0.05; S5 Table). Biological GO pathways that were significantly enriched among females included “regulation of GTPase activity” and “positive regulation of catalytic activity”, which was also significantly enriched among males (S5 Table; FDRs <0.05). No KEGG pathways were significantly enriched in female neonates, potentially due to the smaller number of genes associated with differential methylation in common to both cohorts.

## Discussion

The early life environment during gestation and in infancy is a major factor shaping later life health risk, including susceptibility to the development of obesity. DNA methylation modifications maintain mitotically heritable differences in gene expression in the absence of variations of DNA sequence. Their established role in complex disease and genomic imprinting has led to substantial interest in their potential role as mechanisms mediating the stable programming of health trajectories [55]. Previous studies have largely focused on examining epigenetic differences in offspring by removing data pertaining to sex chromosomes, although there are known sexual dimorphisms in growth and adiposity that occur before birth [8]. In this study, we examined whether DNA methylation modifications associated with maternal obesity in the blood of newborn human infants stratified by offspring sex. We addressed this question by comparing DNA methylation separately in male and female neonatal whole blood from mothers with obesity and compared the identified genes with a large dataset from the ALSPAC cohort. Here we show that DNA methylation profiles in offspring from women with obesity are detectable at birth and they are largely sex-specific.

Using stringent exclusion criteria (excluding common comorbidities associated with maternal obesity such as gestational diabetes and preterm delivery), we found evidence for sex-specific differential DNA methylation and a small of proportion of differentially methylated genes in common to both males and females (~30%) in the OBS cohort. Comparison to the ALSPAC cohort supported these findings, as only a small number of genes were common to both sexes (<10%). Indeed, 75 genes in males and 24 genes in females were common to both cohorts, with only 2 genes common to both sexes across cohorts. Interestingly, *IGF1R* was differentially methylated in males. The methylation status of *IGF1R* was previously shown to respond to diet-induced metabolic stress in animal models and was found to be differentially methylated in lymphocytes in the context of childhood obesity [56, 57]. Epigenetic regulation of *IGF1R* has also been examined in animal models of diabetes. For example, male db/db mice, but not females, show a 7-fold increase in DNA methylation of the Igf1r promotor along with a decrease in levels of Igf1r transcript abundance in skeletal muscle in adulthood, suggesting a sex-specific epigenetic response associated with modifications of gene function in a model of diabetes [58].

Two imprinted genes, namely *GABRB3* and *PRDM16*, also showed differential methylation in both OBS and ALSPAC cohorts in male offspring from mothers with obesity (Table 2; S4 Table). Imprinted genes are implicated in many human disorders, and have important roles in controlling aspects of fetal growth and metabolism [59]. *GABRB3* is associated with the pathogenesis of several disorders including Prader-Willi syndrome, the most common genetic cause of morbid obesity in children [60]. Differential methylation of *PRDM16* is associated with maternal diabetes in blood of children [61] and in umbilical cord tissue [62], and plays an important role in pancreatic development [63]. *PRDM16* is also critical for the differentiation of brown adipose tissue [64], which plays an important role in heat retention and energy expenditure in the first year of life [65] and remains metabolically active into adulthood. Differential methylation of *PRDM16* was observed before gastric bypass and weight loss in adipose tissue, suggesting methylation is modifiable by weight loss [66]. DNA methylation in *PRDM16* was also found to be reversible with neonatal supplementation with resveratrol in male mice [67]. The presence of differential methylation in blood as well as in primary tissues in these studies highlights the potential for *PRDM16* as a biomarker for screening, as well as its potential as a key player in metabolic regulatory mechanisms influenced by maternal obesity. In addition, evidence indicating the responsiveness of PRDM16 to dietary and weight loss intervention suggests it may serve as a molecular target for interventions to mitigate obesity, a hypothesis that deserves further scrutiny.

Among the genes replicated across cohorts in the present study, *MAD1L1* and *PTPRN2* were common to both sexes (Fig 3; Table 2). For *MAD1L1*, a gene involved in control of the cell cycle and tumour suppression [68], four DMRs were found in male neonates and two were found in female neonates. There is evidence that methylation levels at the *MAD1L1* gene locus are responsive to dietary factors (phytoestrogens [69]). In *PTPRN2*, a gene implicated in the secretion of insulin with glucose stimuli [70], six and eight DMRs were found in male (4 DMRs in OBS and 2 in ALSPAC) and female neonates (5 DMRs in OBS and 3 in ALSPAC), respectively. Our findings support previous observations of differential methylation in *PTPRN2* in newborn blood in association with maternal pre-pregnancy BMI, where analyses were performed with both sexes combined [14]. *PTPRN2* encodes a protein that functions as a major islet auto-antigen in type I diabetes [71–73]. Differential methylation in *PTPRN2* in whole blood has been associated with gestational diabetes [74–76], with childhood adiposity [76] as well as childhood obesity [77]. The differential methylation in *PTPRN2* has also been associated with intrauterine condition such as IUGR in blood [78, 79] but also in adults who have experienced famine, *in utero* [80]. These findings provide strong evidence that loci in the fetal *PTPRN2* are likely modifiable with maternal nutrition, and detectable in blood later in life. Our findings showing differential methylation of these genes in blood at birth suggest they may serve as potential biomarkers of increased risk for developing obesity later in life, though this requires additional study.

As discussed above, several genes identified in this study are genes that are differentially methylated at birth, after birth and in disease states, suggesting their potential roles in the long-term programing by aberrant methylation. Notably, accumulating evidence indicates that the methylation status of these genes may be modifiable, suggesting that interventions in the prenatal or early postnatal period may be beneficial in modifying the trajectory of epigenetic modifications that contribute to subsequent development of metabolic disease. While some known risk factors for childhood obesity, such as low socioeconomic status and excessive food consumption, may differ for males and females in the post-natal period [81, 82], our study suggests that additional consideration of the contribution of prenatal factors is warranted.

Sequencing-based approaches continue to expand the scope of epigenetic modifications in genomic elements that can be interrogated by genome-wide methods. Our findings using RRBS suggest that genomic loci outside of promoters and genic elements are associated with differential methylation in the context of exposure to maternal obesity (Fig 2). The role of intergenic DNA methylation modifications associated with environmental exposures is not well understood, but may regulate the activity of distant enhancers and the transcriptional repression of transposable elements [83, 84]. Previous findings using the 450K array, which has limited coverage of intergenic loci, have reported differential methylation at several intergenic regions in relation to BMI in the context of obesity [85, 86]. Our analysis of sequencing data obtained in this study suggest that differential methylation in intergenic regions may be a more prominent feature of DNA modifications associated with maternal obesity than previously thought, at least at CpG-dense loci, and this should be an important consideration for future research.

Our findings should be considered in light of study strengths and limitations, including small sample size. Strengths of this investigation include the use of two independent cohorts for comparison of differentially methylated genes. We acknowledge that the different platforms and associated analytical methods for each cohort (RRBS for the OBS samples, and 450K Illumina microarray for the ALSPAC cohort) are a limitation of this work. However, we note that both platforms are known to show a high correspondence for the detection of differential methylation in regulatory elements associated with genes, particularly when regional-based (DMR) analytical approaches are used [87]. In addition, samples in both cohorts were matched along dimensions known to influence DNA methylation signatures, including exclusions for gestational diabetes and preterm birth. This strategy avoided confounds of pregnancy complications shown to affect DNA methylation, but imposed limitations on the number of samples available. Thus, our findings should be considered preliminary. An additional limitation of this study concerns the use of whole blood, which may be less informative of metabolic dysregulation compared to muscle or fat. However, we note that blood constitutes a more readily accessible tissue, particularly in large-scale studies and studies in early life, and is known to reflect metabolic and immune pathways active in the context of obesity [88, 89]. Importantly, the hypothesis that genes associated with maternal obesity were differentially methylated at birth was supported in both cohorts. Additional work is needed to understand the potential relationship between these early methylation modifications detected at birth and later outcomes.

In animal studies, maternal obesity during pregnancy has been shown to result in a number of poor health indicators in offspring [90, 91]. Our results support the association of maternal obesity and biological signatures in humans at birth. There is a need to identify biomarkers prior to the emergence of poor health outcomes, when interventions that support mothers and their families are likely to be most effective. Ten per cent of school-aged children are estimated to be either overweight or obese worldwide [92]. These children have a significant likelihood of developing type 2 diabetes, heart disease and other co-morbidities before or during their early adulthood. Better prediction of vulnerable children and help to support optimal health will require a proven understanding of developmental mechanisms leading to adverse health outcomes.

In conclusion, maternal obesity is associated with DNA methylation modifications at loci in whole blood in a manner that is sex-specific. The present results support growing evidence indicating that sexual dimorphism is an important feature of the response to maternal obesity during early development. Our results support the feasibility of assessing sex-specific differences in DNA methylation in the very early post-partum period associated with the effect of maternal obesity. Features of DNA methylation modifications, including its relative stability in comparison to RNA and its potential as a mediator of adverse environmental exposures, have made them attractive candidates for the study of the Developmental Origins of Health and Disease (DOHaD). As there is accumulating evidence suggesting the mediating role of DNA methylation modifications in sex-specific childhood growth trajectories [93], the sites of DNA methylation modifications identified in this study may represent candidate loci for future studies including interventions targeting metabolic processes and diet. The identification of DNA methylation modifications in peripheral tissues such as blood should advance translational studies of the impact of maternal obesity on epigenetics and subsequent human disease.

## Supporting information

S1 Fig Histograms of the percent methylation distribution for males (A) and females (B) in the OBS cohort. X axis shows percent methylation per CpG site and y axis shows the frequency. C: Neonates from non-obese control mothers (n = 10/sex), O: Neonates from mothers with obesity (n = 10/sex).
(PDF)

S1 Table Correlation matrix of DNA methylation between samples for males (A) and females (B) in the OBS discovery cohort. C: neonates of non-obese control mothers (n = 10/sex), O: neonates of mothers with obesity (n = 10/sex).
(XLSX)

S2 Table Full list of DMRs, ranked by absolute methylation difference percentage for male (A) and female (B) neonates in the OBS discovery cohort. (XLSX)

S3 Table Full list of DMRs, ranked by absolute methylation difference percentage for male (A) and female (B) neonates in the ALSPAC comparison cohort. (XLSX)

S4 Table Full list of genes associated with DMRs in common between OBS and ALSPAC cohorts for male (A) and female (B) neonates. (XLSX)

S5 Table Full list of Gene Ontology pathways for biological process based on genes in common between OBS and ALSPAC cohorts in male (A) and female (B) neonates. (XLSX)
