# Supplementary material for: DNA methylation profiles in the blood of newborn term infants born to mothers with obesity
Source: PLoS One. 2022 May 2;17(5):e0267946. doi: 10.1371/journal.pone.0267946 (PMC9060365; doi:10.1371/journal.pone.0267946)
Supplement: S1 Fig — X axis shows percent methylation per CpG site and y axis shows the frequency. C: Neonates from non-obese control mothers (n = 10/sex), O: Neonates from mothers with obesity (n = 10/sex). (PDF) [file pone.0267946.s001.pdf]

S1 Fig. Histograms of the percent methylation distribution for males (A) and females (B) in the OBS cohort. X axis shows percent methylation per CpG site and y axis shows the frequency. C: Neonates from non-obese control mothers (n=10/sex), O: Neonates from mothers with obesity (n=10/sex).

A (Males)

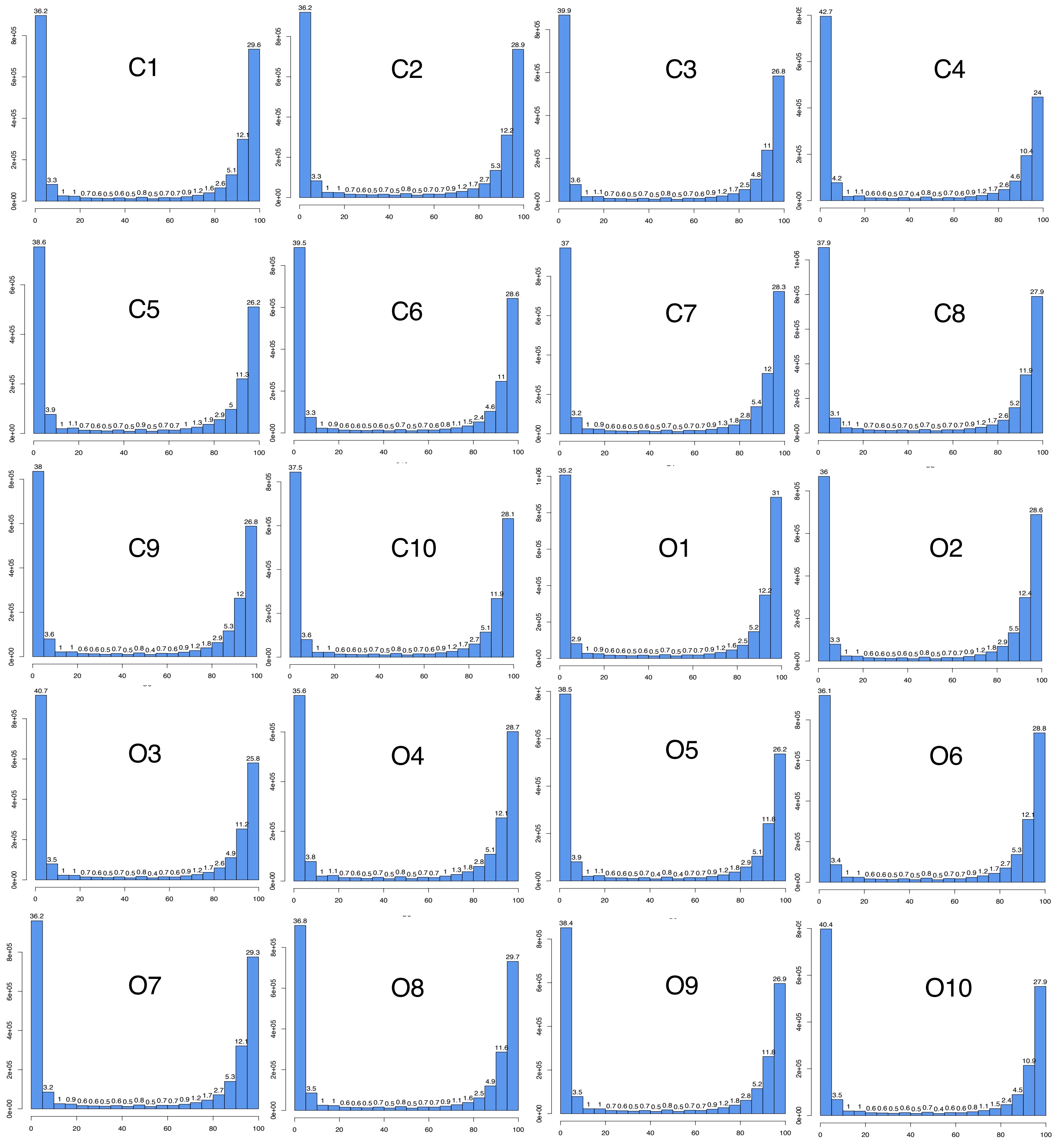

B (Females)

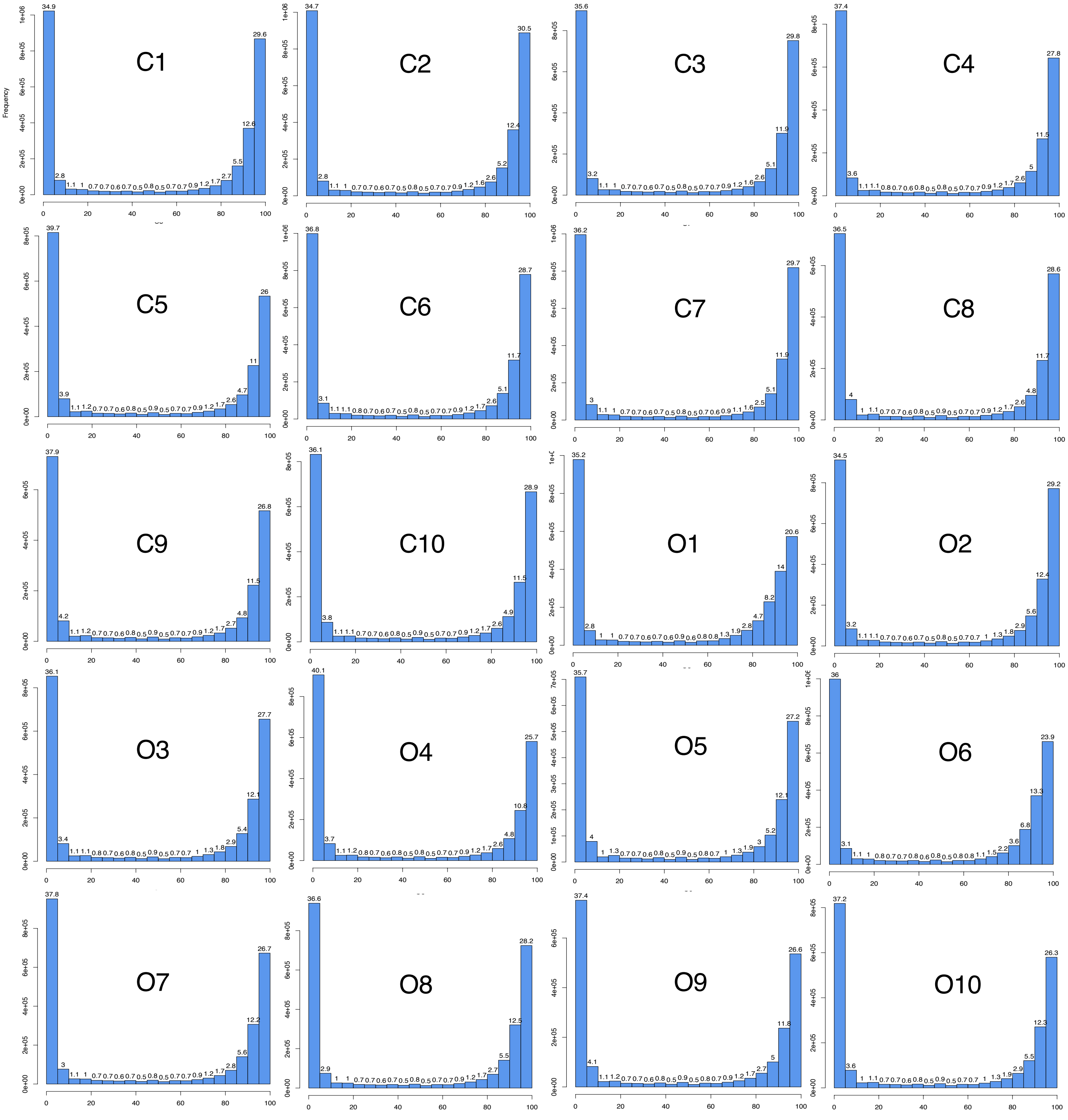

C1
C2
C3
C4
C5
C6
C7
C8
C9
C10
O1
O2
O3
O4
O5
O6
O7
O8
O9
O10
Frequency
